# PD11 Cost-Effectiveness Analysis Of Inclisiran For Treating Primary Hypercholesterolemia And Mixed Dyslipidemia In Singapore

**DOI:** 10.1017/S0266462324002794

**Published:** 2025-01-07

**Authors:** Yan Ling Lim, Ru San Tan, Kian Keong Poh, Xiao Jun Wang

## Abstract

**Introduction:**

Inclisiran is approved in Singapore as an add-on to maximally tolerated statins for the treatment of primary hypercholesterolemia or mixed dyslipidemia. This study evaluated the cost effectiveness of inclisiran compared with standard care (SC), evolocumab, or alirocumab to treat primary hypercholesterolemia and mixed dyslipidemia.

**Methods:**

A lifetime Markov model was used to evaluate the cost effectiveness of inclisiran as an add-on to SC (inclisiran+SC) versus SC alone, evolocumab+SC, or alirocumab+SC in four subpopulations: atherosclerotic cardiovascular disease (ASCVD), secondary prevention of heterozygous familial hypercholesterolemia (HeFH), primary prevention of HeFH, and primary prevention in patients with an elevated risk of ASCVD Baseline cardiovascular event risks were estimated from databases and published literature from the Netherlands and the UK. Efficacy data were obtained from the ORION trials and other comparative trials. Costs were obtained from public healthcare institutions and local publications. A willingness-to-pay (WTP) threshold of SGD45,000 (USD33,280) per quality-adjusted life-year (QALY) was selected, and a one-way deterministic sensitivity analysis (DSA) was performed.

**Results:**

Inclisiran+SC resulted in higher QALYs and higher total costs than SC alone in all four subpopulations, with incremental cost-effectiveness ratios (ICERs) ranging from SGD34,654 to SGD163,158 (USD25,630 to USD120,673) per QALY gained. At the selected WTP threshold, inclisiran+SC was cost effective, compared with SC, in patients with ASCVD and for secondary prevention of HeFH. Compared with evolocumab+SC and alirocumab+SC, inclisiran+SC achieved higher total QALYs at a lower total cost in all four subpopulations. The ICER was most sensitive to the price and efficacy of inclisiran and the rate ratios translating reductions in low-density lipoprotein cholesterol levels to the risk of cardiovascular death.

**Conclusions:**

Inclisiran+SC resulted in greater QALYs and higher costs, compared with SC alone, and higher QALYs at lower costs, compared with evolocumab+SC and alirocumab+SC, in adults with primary hypercholesterolemia and mixed dyslipidemia. At the selected WTP threshold, inclisiran+SC was cost effective, compared with SC alone, in patients with ASCVD and for secondary prevention of HeFH. These findings can help inform future funding decisions in Singapore.

